# Single-cell multiomics: technologies and data analysis methods

**DOI:** 10.1038/s12276-020-0420-2

**Published:** 2020-09-15

**Authors:** Jeongwoo Lee, Do Young Hyeon, Daehee Hwang

**Affiliations:** School of Biological Sciences, Seoul National University, Seoul, 08826 Republic of Korea

**Keywords:** Transcriptomics, Proteomics, Data integration

## Abstract

Advances in single-cell isolation and barcoding technologies offer unprecedented opportunities to profile DNA, mRNA, and proteins at a single-cell resolution. Recently, bulk multiomics analyses, such as multidimensional genomic and proteogenomic analyses, have proven beneficial for obtaining a comprehensive understanding of cellular events. This benefit has facilitated the development of single-cell multiomics analysis, which enables cell type-specific gene regulation to be examined. The cardinal features of single-cell multiomics analysis include (1) technologies for single-cell isolation, barcoding, and sequencing to measure multiple types of molecules from individual cells and (2) the integrative analysis of molecules to characterize cell types and their functions regarding pathophysiological processes based on molecular signatures. Here, we summarize the technologies for single-cell multiomics analyses (mRNA-genome, mRNA-DNA methylation, mRNA-chromatin accessibility, and mRNA-protein) as well as the methods for the integrative analysis of single-cell multiomics data.

## Single-cell profiling: understanding disease at the cellular level

The expansion of single-cell profiling technologies will provide unprecedented insights into the molecular mechanisms inherent in disease. Novel technologies known collectively as ‘single-cell multiomics’ enable systematic, high-resolution profiling of DNA, RNA and proteins in individual cells. This provides valuable data about gene regulation and molecular populations, and cellular processes during disease development and progression. Daehee Hwang and co-workers at Seoul National University, Seoul, South Korea, reviewed existing single-cell multiomics technologies and highlighted ways to integrate the data generated. Analytical features of multiomics allow scientists to isolate, sequence and label (or ‘barcode’) multiple molecules in single cells. Different sequencing techniques can be used for different purposes, such as exploring gene mutation coverage or measuring RNA transcripts. Combining these sequencing data will help identify links between significant features during disease.

## Introduction

Recent advances in single-cell isolation and barcoding technologies have enabled DNA, mRNA, and protein profiles to be measured at a single-cell resolution. Various experimental protocols have been developed and applied to diverse cellular systems to demonstrate the power of single-cell level analyses^1–4^. For example, Tirosh et al.^5^ applied single-cell RNA sequencing (scRNA-seq) to human melanoma and identified two groups of malignant cells with high expression of the microphthalmia-associated transcription factor (*MITF*) gene: a master melanocyte transcriptional regulator group (MITF-high cells) and a group expressing the AXL gene conferring resistance to targeted therapies (AXL-high cells). Although bulk analysis showed that each tumor could be classified as MITF-high or AXL-high, the single-cell analysis further revealed that every tumor contained both groups of malignant cells, but the MITF-high tumors harbored a subpopulation of AXL-high cells that were undetectable through bulk analysis and vice versa. Moreover, Villani et al.^6^ clustered human blood dendritic cells (DCs) and monocytes using scRNA-seq and identified a subpopulation of DCs with a potent T cell activation ability. These studies demonstrate that single-cell analyses provide unique insights into cell subpopulations and their functions associated with pathophysiological processes.

Multiomics analyses at the bulk tumor level have been reported to provide a comprehensive understanding of cellular processes through the integration of different types of molecular data (e.g., data on mutations, mRNAs, proteins, and metabolites). For example, proteogenomic analyses have been applied to colorectal^7,8^, ovarian^9,10^, breast^11,12^, and gastric cancers^13^. Mun et al.^13^ identified correlations between somatic mutations (e.g., nonsynonymous somatic mutations in the ARID1A gene, a component of SWI/SNF chromatin remodeling complexes) and altered signaling pathways (e.g., PI3K-AKT and MAPK signaling), which facilitate the interpretation of the functional associations of somatic mutations and signaling pathways in gastric cancers. Moreover, they found that patient subtypes identified on the basis of mRNA expression patterns could be further divided according to protein abundance and/or phosphorylation data, providing detailed molecular signatures for immunogenic and invasive diffuse gastric cancers. Other integrative analyses of mRNA data with DNA methylation, histone modification, microRNA and/or mutation data have also been reported^14–17^. These multiomics studies demonstrate that integrative analyses of different types of omics data can provide more comprehensive insights into tumor biology than a single type of omics data alone due to their complementary nature.

The advantage of this approach has prompted the development of single-cell multiomics technologies. Various experimental protocols for single-cell multiomics analysis (e.g., mRNA-DNA methylation and mRNA-protein) have been developed and applied to examine cell type-specific gene regulation. Gaiti et al.^18^ integrated single-cell transcriptome and DNA methylome data and identified a lineage tree of human chronic lymphocytic leukemia (CLL) after drug (ibrutinib) treatment and its link to the transcriptional transition after therapy. They first used epigenome data to construct a lineage tree for CLL cells based on stochastic DNA methylation changes, referred to as epimutations, and found that different CLL lineages were preferentially affected by ibrutinib and expelled from the lymph nodes after treatment. By projecting the transcriptome data onto the lineage tree, they further found that the cells preferentially affected by ibrutinib showed upregulation of genes involved in cell cycle and Toll-like receptor signaling. Jia et al.^19^ also integrated single-cell transcriptome and chromatin accessibility data to study the developmental trajectories of mouse embryonic cardiac progenitor cells and identified marker genes linking transcriptional and epigenetic regulation during development. Therefore, single-cell multiomics analysis can provide more comprehensive insights into cell type-specific gene regulation than single-cell mono-omics analysis.

**Fig. 1:**
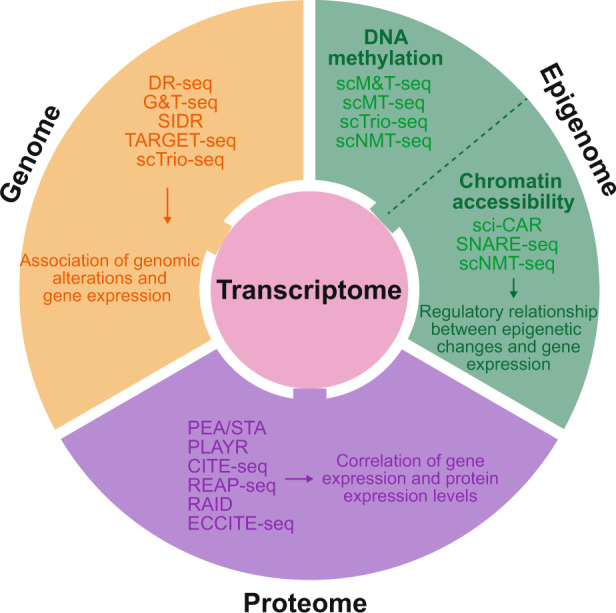
An overview of single-cell multiomics sequencing technologies. Single-cell multiomics sequencing technologies and the expected outcomes are illustrated. Technologies that measure more than two types of data are included in multiple categories (e.g., scTrio-seq in transcriptome-genome and transcriptome-DNA methylation categories).

The core components of single-cell multiomics analysis are (1) technologies for single-cell isolation, barcoding, and sequencing, to measure multiple types of molecules from the same cells, and (2) integrative analysis of the molecules measured at the single-cell level, to identify cell types and their functions related to pathophysiological processes based on the molecular signatures. Here, we first review the technologies used in single-cell multiomics analyses, mainly focusing on mRNA-genome, mRNA-DNA methylation, mRNA-chromatin accessibility, and mRNA-protein data (Fig. 1 and Table 1). By presenting representative applications of these technologies, we illustrate the expected outcomes from the integrative analysis of multiple types of data, including associations of genomic alterations and gene expression, regulatory relationships between epigenetic changes and gene expression, and correlations between mRNA and protein expression (Fig. 1). Finally, we summarize the methods for the integrative analysis of single-cell multiomics data.

**Table 1:** Single-cell multiomics technologies.

Category of multiomics	Methods	Cell isolation and DNA/RNA/protein separation	Molecules measured	Cell throughput	Automation	Notes	Refs
Genome-transcriptome	G&T-seq	Flow cytometry (cell isolation); and bead-based separation (DNA and polyadenylated mRNA)	gDNA and polyadenylated mRNA (whole cell)	Medium	Yes		^23^
	DR-seq	Cell picking by pipette (cell isolation); and preamplification and tagging of DNA and RNA followed by splitting	gDNA and polyadenylated mRNA (whole cell)	Low	No		^24^
	SIDR	Microplate-based cell isolation; and separation of nucleus and cytoplasm using hypotonic lysis	gDNA and polyadenylated cytosolic RNA	Low			^32^
	TARGET-seq	FACS (cell isolation); and reverse transcription and amplification followed by library preparation	Targeted gDNA, polyadenylated mRNA, and targeted mRNA (whole cell)	High			^33^
Genome-transcriptome- DNA methylome	scTrio-seq	Cell picking by pipette; and centrifugation-based separation of nucleus and cytoplasm followed by bisulfite treatment	CNVs (computational inference), gDNA methylation, and polyadenylated cytosolic RNA	Low	No	Bisulfite treatment	^22^
Transcriptome-DNA methylome	scM&T-seq	Flow cytometry (cell isolation); and bead-based separation of DNA and polyadenylated mRNA followed by bisulfite treatment	gDNA methylation and polyadenylated RNA (whole cell)	Medium	Yes	Bisulfite treatment	^39^
	scMT-seq	Micropipetting for isolation of single nuclei.	gDNA methylation and polyadenylated cytosolic RNA	Low	Partial	Bisulfite treatment	^40^
Transcriptome-chromatin accessibility	sci-CAR	Combinatorial indexing; and lysate splitting followed by library preparation	Chromatin and polyadenylated nuclear RNA	High			^49^
	SNARE-seq	Microfluidic channels (cell isolation); and open chromatin tagmentation followed by dual-omics capture	Chromatin and polyadenylated nuclear RNA	High			^50^
	Paired-seq	Combinatorial indexing; and preamplification followed by splitting and enzymatic digestion	Chromatin and polyadenylated nuclear RNA	High			^52^
Transcriptome-DNA methylome-chromatin accessibility	scNMT-seq	FACS (cell isolation); GpC labeling; and bead-based separation of nucleus and cytoplasm followed by bisulfite treatment	gDNA methylation, chromatin, and polyadenylated cytosolic RNA,	Medium	Partial	Bisulfite treatment	^51^
Transcriptome-proteome	PEA/STA	Microfluidic channels (cell isolation); and reverse transcription of PEA probe and RNA followed by targeted amplification	Targeted RNA and targeted proteins	Medium	Yes		^55^
	PLAYR	Flow or mass cytometry (cell isolation); and detection of amplified product of PLAYR probe pair and antibody staining	Targeted cytosolic RNA and targeted proteins	High	No	Cell fixation and permeabilization	^56^
	CITE-seq	Drop-seq and 10X Genomics platform (cell isolation); and reverse transcription of mRNA and antibody-derived oligonucleotides followed by separation of libraries	Polyadenylated RNA and targeted cell surface proteins	High	No		^57^
	REAP-seq	10x Genomics Chromium (cell isolation); and reverse transcription of mRNA and antibody-derived oligonucleotides followed by separation of libraries	Polyadenylated RNA and targeted cell surface proteins	High	No		^58^
	RAID	Plate-based cell isolation; and cell crosslinking and immunostaining with RNA-barcoded antibodies	Polyadenylated RNA and targeted intracellular proteins	High		Cell fixation and permeabilization	^59^
Transcriptome-proteome-clonotypes-CRISPR perturbations	ECCITE-seq	10X Genomics platform (cell isolation); and capture of mRNA, sgRNA, and antibody-derived oligonucleotides followed by separation of libraries	Polyadenylated RNA, sgRNA, and targeted cell surface proteins	High		Compatible with existing CRISPR guide libraries	^61^

Cell throughput is categorized as low, medium, or high based on the number of cells applied in each reference. Low, fewer than 50 cells; Medium, from 50 to 200 cells; High, more than 200 cells.

*G&T-seq* genome and transcriptome sequencing, *DR-seq* gDNA-mRNA sequencing, *SIDR* simultaneous isolation of genomic DNA and total RNA, *scTrio-seq* single-cell triple omics sequencing, *scM&T-seq* single-cell methylome and transcriptome sequencing, *scMT-seq* single-cell methylome and transcriptome sequencing, *sci-CAR* single-cell combinatorial indexing chromatin accessibility and mRNA, *SNARE-seq* single-nucleus chromatin accessibility and mRNA expression sequencing, *Paired-seq* parallel analysis of individual cells for RNA expression and DNA accessibility by sequencing, *scNMT-seq* single-cell nucleosome, methylation and transcription sequencing, *PEA/STA* proximity extension assay/specific (RNA) target amplification, *PLAYR* proximity ligation assay for RNA, *CITE-seq* cellular indexing of transcriptomes and epitopes by sequencing, *REAP-seq* RNA expression and protein sequencing assay, *RAID* single-cell RNA and immuno-detection, *CRISPR* clustered regularly interspaced short palindromic repeats, *ECCITE-seq* expanded CRISPR-compatible cellular indexing of transcriptomes and epitopes by sequencing, *FACS* fluorescence-activated cell sorting, *Drop-seq* droplet-sequencing, *sgRNA* single-guide RNA, *CNV* copy number variation.

## Cell isolation and barcoding

For single-cell multiomics analysis, it is essential to isolate multiple types of molecules from the same cells, which involves (1) the isolation of single cells and (2) the subsequent barcoding of multiple types of molecules. The isolation of single cells begins with the mechanical or enzymatic dissociation of viable cells followed by capturing single cells from the dissociated cell suspension. Several capture methods used for single-cell mono-omics analysis are commonly employed in single-cell multiomics analysis, including (1) low-throughput methods to capture tens or hundreds of cells, including laser capture microdissection^20^ and robotic micromanipulation^21^, and (2) high-throughput methods to capture tens of thousands of cells, including fluorescence-activated cell sorting (FACS) followed by plate-based isolation and the use of microfluidic platforms with microfluidic channels and reaction chambers or nanowells^4^. Low-throughput methods retain spatial information on the isolated cells, while this information is lost under high-throughput methods.

Multiple types of molecules are then isolated from the individual captured cell. Genomic DNA (gDNA) is located in the nucleus, while the majority of mRNAs are contained in the cytosol. After treatment with a plasma membrane-selective lysis buffer, the nuclei are separated from the cytoplasm by centrifugation^22^. gDNA is isolated from the nuclei, while mRNAs are isolated from the cytoplasm, resulting in the loss of mRNAs located in the nucleus. In an alternative method, oligo-dT-coated magnetic beads are used to selectively capture mRNAs, and the pull-down of these beads using a magnet allows the mRNAs to be separated from gDNA^23^. Under these methods, different barcodes (cell- and molecule-identifying barcodes) are used for the separated gDNA and mRNA to distinguish gDNA and mRNA from each other. However, the separation process can lead to sample loss. To resolve this problem, an alternative strategy that does not require separation was developed^24^. Under this method, mRNAs are reverse transcribed (RT) with no separation after cell lysis using poly-dT primers, producing single-stranded cDNA. gDNA and cDNA are simultaneously amplified via quasilinear whole-genome amplification with primers similar to multiple annealing and looping-based amplification cycles (MALBAC) adapters. After the product is split into two portions, gDNA is amplified from one half of the product by polymerase chain reaction (PCR), and cDNAs are amplified from the other half by in vitro transcription.

Additional precautions must be taken sufficiently to measure multiple types of molecules from the same cell. Clinical samples are often flash-frozen or embedded in paraffin, and the freezing process disturbs the cytoplasmic membrane but not the nuclear membrane. For these samples, the single-cell multiomics analysis of gDNA and nuclear mRNA after the isolation of single-cell nuclei is still possible, but the analysis of cytosolic mRNAs can lead to misleading conclusions^25^. For fresh tissues, however, prolonged exposure to dissociation enzymes or extensive mechanical mincing can result in the degradation or perturbation of mRNAs and proteins, respectively^26^.

## Integrative analysis of genome and transcriptome data

Various sequencing methods for single-cell multiomics analysis have been adopted from those developed for single-cell mono-omics analysis. Single-cell whole-genome sequencing (scWGS) methods include multiple displacement amplification (MDA)^27^, MALBAC^28^, and PicoPLEX (Rubicon Genomics PicoPLEX Kit). Single-cell RNA sequencing (scRNA-seq) methods include Quartz-seq^29^, switching mechanism at the 5′ end of the RNA transcript (Smart-seq)^30^, and cell expression by linear amplification and sequencing (CEL-seq)^31^. These methods involve different strategies to achieve different purposes. Quartz-seq measures the 3′ end of transcripts, while Smart-seq measures full-length transcripts, and CEL-seq barcodes and pools samples before the linear amplification of mRNAs to multiplex single-cell samples.

**Fig. 2:**
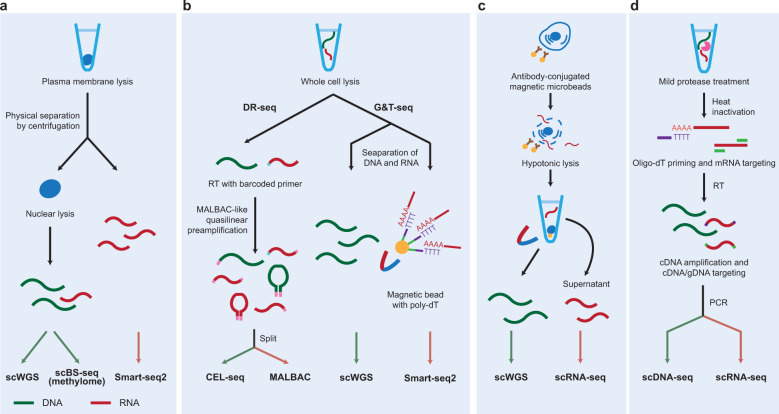
Single-cell multiomics sequencing protocols for the integrative analyses of the genome and transcriptome. Protocols for the isolation of single cells or nuclei and the barcoding of gDNA and mRNAs are shown for five types of multiomics analyses of the genome and transcriptome: scTrio-seq (**a**), DR-seq and G&T-seq (**b**), SIDR (**c**), and TARGET-seq (**d**). Blue solid circles, nucleus; blue dotted line, permeabilized membrane; red and green lines, mRNA and gDNA, respectively; yellow solid circles, beads; Y shapes, antibodies; magenta and green fragments, barcodes or primers; and U shapes, magnets. See text for the definitions of the abbreviations.

Several approaches for single-cell multiomics analyses of the genome and transcriptome have been developed, including single-cell triple omics sequencing (scTrio-seq)^22^, genome and transcriptome sequencing (G&T-seq)^23^, gDNA-mRNA sequencing (DR-seq)^24^, simultaneous isolation of genomic DNA and total RNA (SIDR)^32^, and TARGET-seq^33^. The characteristics of these technologies are summarized in Table 1. scTrio-seq involves the physical separation of the cytoplasm (cytoplasmic mRNAs) and nucleus (gDNA) from the same single cells by centrifugation (Fig. 2a). The separated gDNA and mRNAs are then independently amplified and sequenced using scWGS protocols (e.g., MDA or PicoPLEX) and Smart-seq2, respectively. G&T-seq separates poly-A-tailed mRNAs from gDNA using oligo-dT-coated magnetic beads as described above. The separated mRNAs and gDNA are then sequenced using Smart-seq2 and scWGS protocols, respectively (Fig. 2b, right). DR-seq involves the aforementioned simultaneous MALBAC-like quasilinear preamplification of gDNA and cDNA with no separation of gDNA and mRNA (Fig. 2b, left). After the preamplified gDNA and cDNA are split into two fractions, scRNA-seq and scWGS are separately performed for the two fractions using CEL-seq and MALBAC, respectively. However, DR-seq presents limited options under the WGS method^24^ and is unable to sequence full-length transcripts, precluding the detection of splicing variants and fusion transcripts^34^. Another method referred to as SIDR was developed. Under this method, cells are incubated with antibody-conjugated magnetic microbeads, and bead-labeled single cells are sorted into a 48-well microplate (Fig. 2c). Hypotonic lysis is then applied to release cytosolic RNAs from the captured single cells while preserving nuclear lamina integrity, followed by the isolation of the RNA-containing supernatant from the nucleus-containing cell lysate. The TARGET-seq approach, which can improve the coverage of key mutations, was developed recently. Under this method, mild protease digestion is used to improve the release of gDNA and mRNAs during cell lysis; heat inactivation of the protease is performed to avoid the inhibition of RT and PCR; and RT and PCR amplification is followed by scRNA and targeted scDNA-seq, respectively (Fig. 2d).

Several studies using these methods have reported that genomic alterations are closely correlated with the transcription levels of the genes in the altered regions of the genome. For example, using G&T-seq, Macaulay et al.^23^ identified a subpopulation of HCC38-BL cells exhibiting trisomy of chromosome 11. The expression of genes on chromosome 11 was higher in this subpopulation than in diploid cells. Genomic imbalances on chromosome 16 were also found to be consistent with the changes in the expression of genes in the region with the imbalance. Moreover, after applying DR-seq to SK-BR-3 breast cancer cells, Dey et al.^24^ compared copy number variations (CNVs) with mRNA expression levels and observed a monotonic increase in the mean expression of genes within the regions with increased copy numbers across the single cells. Using TARGET-seq, Rodriguez-Meira et al.^33^ also found aberrant expression of oncogenes (e.g., MYCN, TP53, and PPP2R5A), genes related to hedgehog and Wnt signaling, or interferon-associated genes in JAK2V617F-mutated hematopoietic stem and progenitor cells from patients with myeloproliferative neoplasms. All these data demonstrate the correlation of genomic alterations (e.g., CNVs or mutations) with gene expression at the genome level in single cells.

## Integrative analysis of the transcriptome with epigenome data

**Fig. 3:**
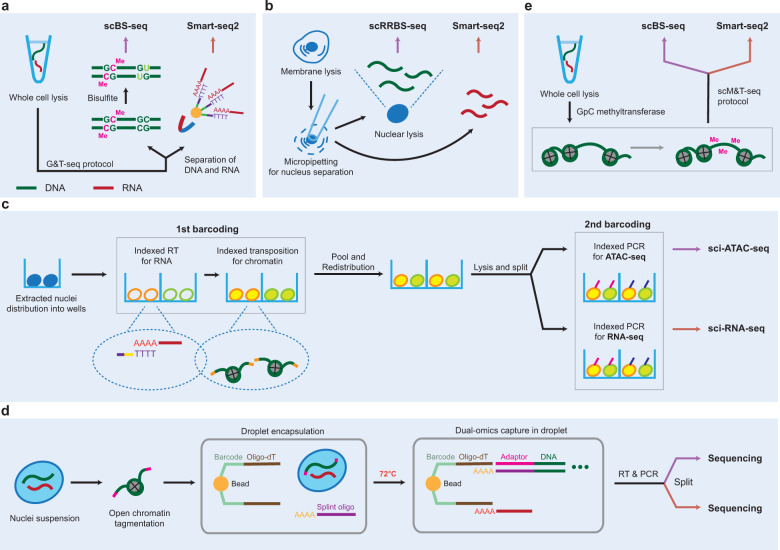
Single-cell multiomics sequencing protocols for the integrative analysis of the transcriptome and epigenome. Protocols for the isolation of single cells or nuclei and the barcoding of gDNA and mRNAs are shown for five types of multiomics analyses of the transcriptome and epigenome: scM&T-seq (**a**) and scMT-seq (**b**) for DNA methylation and sci-CAR (**c**), SNARE-seq (**d**), and scNMT-seq (**e**) for chromatin accessibility. Gray cross, nucleosome; Me, CH_3_. In **c**, the colors of the border line, inside, and tip of the circles distinguish the barcodes of mRNAs, accessible DNA fragments, and indexed PCR, respectively. See the legend in Fig. 2 for the other symbols and the text for the definitions of the abbreviations.

DNA methylation, histone modifications (e.g., methylation and acetylation), and chromatin accessibility collectively contribute to gene expression and have been shown to be measured at a single-cell resolution. Single-cell bisulfite sequencing (scBS-seq) methods for measuring the single-cell DNA methylome include single-cell reduced representative bisulfite sequencing (scRRBS)^35^, single-cell whole-genome bisulfite sequencing (scWGBS)^36^, single-nucleus methylcytosine sequencing (snmC-seq)^37^, and single-cell combinatorial indexing for methylation (sci-MET)^38^. The first single-cell multiomics analysis of the DNA methylome and transcriptome was performed via the scM&T-seq (single-cell methylome and transcriptome sequencing) approach, in which the G&T-seq procedure is used to isolate and amplify gDNA and RNA from the same single cell, and scBS-seq is applied to the amplified gDNA to generate DNA methylome data^39^ (Fig. 3a). Hu et al.^40^ developed single-cell methylome and transcriptome sequencing (scMT-seq), in which micropipetting is used to isolate the nuclei from the lysates of single cells, and performed scRRBS and a modified Smart-seq2 procedure to generate DNA methylome and transcriptome data, respectively (Fig. 3b). Moreover, scTrio-seq, which profiles the genome, methylome, and transcriptome, uses scRRBS to generate DNA methylatome data^22^. However, one limitation of these methods is the loss of information due to DNA degradation caused by bisulfite treatment^41^. The characteristics of these technologies are summarized in Table 1.

Droplet-based chromatin immunoprecipitation (Drop-ChIP) sequencing has been developed to measure modifications of histone proteins (histone H3 di- and tri-methylation) at a single-cell resolution^42^. Under this method, microfluidic devices are used to encapsulate a single cell in a droplet with a lysis detergent and micrococcal nuclease, generating mono-, di-, or trinucleosomes. These nucleosome droplets are then merged one by one with a droplet containing a cell-specific barcode, generating barcoded chromatin fragments. ChIP-seq can then be performed on these pooled fragments to identify histone modification sites. However, this method produces a low coverage of the DNA methylome (~800 peaks per cell). Single-cell multiomics analysis using Drop-ChIP has rarely been explored. The profiling of open chromatin (e.g., promoters and enhancers) can be used to predict operative transcription factors by footprinting analysis^43^. Single-cell chromatin accessibility methods include single-cell DNase sequencing (scDNase-seq)^44^, single-cell combinatorial indexing assay for transposase-accessible chromatin with sequencing (sci-ATAC-seq)^45^, single-cell assay for transposase-accessible chromatin using sequencing (scATAC-seq)^46^, nucleosome occupancy and methylation sequencing (NOMe-seq)^47^, and single-cell micrococcal nuclease sequencing (scMNase-seq)^48^. Based on these methods, several strategies for the multiomics analysis of chromatin accessibility and transcriptomes have been developed, including single-cell combinatorial indexing chromatin accessibility and mRNA (sci-CAR)^49^, single-nucleus chromatin accessibility and mRNA expression sequencing (SNARE-seq)^50^, and single-cell nucleosome, methylation and transcription sequencing (scNMT-seq)^51^.

The sci-CAR method measures both open chromatin sites and mRNA levels from the same single nuclei using plate-based single-nucleus isolation and combinatorial indexing^49^. This method barcodes nuclear mRNAs using indexed RT and open chromatin sites via indexed transposition with barcode-carrying transposases (Fig. 3c). All nuclei are then pooled, redistributed, and lysed. After the nuclear lysate is split into two portions, a second barcode is added with indexed PCR for RNA-seq in one half and with indexed PCR for ATAC-seq in the other half. Combinatorial barcoding enables mRNAs and open chromatin from single nuclei to be distinguished. Recently, Zhu et al.^52^ developed parallel analysis of individual cells for RNA expression and DNA accessibility by sequencing (Paired-seq), which is similar to sci-CAR-seq but involves the use of restriction enzymes at the final step. SNARE-seq is another method for profiling open chromatin and mRNAs from the same single nuclei using a microdroplet platform and barcoded beads^50^. The protocol for this method begins with the isolation of single nuclei and open chromatin tagmentation in the isolated nuclei using a transposase (Fig. 3d). The tagmented nuclei are then encapsulated in a droplet including both an oligo-dT-containing barcoded bead and a splint oligonucleotide, which links the tagmented gDNA fragments to the bead, enabling the bead to capture both mRNAs and open chromatin fragments. After mRNAs and gDNA fragments are released from the bead by heating, RT and PCR amplification are performed to generate a library of cDNA and open chromatin gDNA fragments. Moreover, scNMT-seq^51^ was developed to profile the nucleosome, DNA methylome, and transcriptome from the same single cells by combining scM&T-seq and NOMe-seq (Fig. 3e). The characteristics of these technologies are summarized in Table 1.

Several studies using these methods have reported that DNA methylation differences are correlated with variation in gene transcription across single cells. For example, using scM&T-seq, Angermueller et al.^39^ found that regions of low methylation showed high variance in methylation levels, consistent with their roles as distal regulatory elements that control gene expression. Using scM&T-seq, Hernando-Herraez et al.^53^ also identified a link between epigenetic and transcriptional signatures associated with the aging of tissue-specific mouse stem cells. Moreover, using scMT-seq, Hu et al.^40^ found that non-CpG island promoters showed variable CpG enrichment, contributing to methylome heterogeneity among dorsal root ganglion single cells. They further found that transcript levels were positively correlated with gene body methylation but negatively correlated with promoter methylation and found a correlation between allelic gene body methylation and allelic gene expression in single cells^40,54^. Moreover, using scNMT-seq, Clark et al.^51^ identified dynamic coupling of the nucleosomes, DNA methylome, and transcriptome in single mouse embryonic stem cells during their differentiation. All these data demonstrate the links between the epigenome and gene expression at the genome level in single cells.

## Integrative analysis of transcriptome and proteome data

**Fig. 4:**
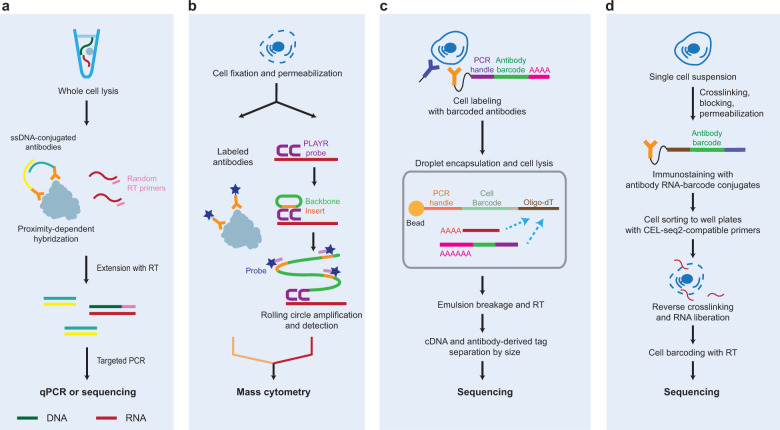
Single-cell multiomics sequencing protocols for integrative analyses of the transcriptome and proteome. Protocols for the isolation of single cells and the barcoding of mRNAs and proteins are shown for four types of multiomics analyses of the transcriptome and proteome: PEA/STA (**a**), PLAYR (**b**), CITE-seq (**c**), and RAID (**d**). Green-blue line, single-stranded DNA (ssDNA) oligos conjugated to antibodies; rotated U shapes, PLAYR probes; green-orange circle, backbone-insert oligos; and DNA fragments containing stars, isotope-labeled probes. See the legend of Fig. 2 for the other symbols and the text for the definitions of the abbreviations.

Despite the biological importance of proteins, the number of proteins that can be measured by single-cell proteome profiling is limited because proteins cannot be amplified, unlike DNA and mRNA. Several methods that can measure both the transcriptome and proteome of a single cell have been developed, including proximity extension assay/specific RNA target amplification (PEA/STA)^55^, proximity ligation assay for RNA (PLAYR)^56^, cellular indexing of transcriptomes and epitopes by sequencing (CITE-seq)^57^, and RNA expression and protein sequencing assay (REAP-seq)^58^ (Table 1). Under the PEA/STA method, proximity extension assay (PEA)-tagged antibody pairs are used for the proximity-dependent hybridization of DNA oligos ligated to the antibody pairs, which convert proteins into DNA oligos, and RT is carried out for mRNAs using random RT primers to generate cDNAs (Fig. 4a). Both DNA oligos and cDNAs are then amplified by PCR and quantified using quantitative PCR or sequencing. The PLAYR method labels proteins with antibodies containing elemental isotopes and uses PLAYR probes that bind to mRNAs. Adjacent PLAYR probe pairs provide a docking site for RNA-specific insert-backbone oligos, and they are then ligated to isotope-labeled probes through rolling circle amplification, which converts mRNA levels into isotope label levels (Fig. 4b). Subsequently, the levels of isotope-labeled mRNAs and proteins are measured by mass cytometry.

Recently, CITE-seq and REAP-seq have been developed to detect both cell surface proteins and mRNAs using oligonucleotide-labeled antibodies. For example, in a single-cell suspension, CITE-seq first tags the cells expressing target proteins on the surface using target-specific antibodies conjugated with DNA oligos containing PCR handles, antibody-identifying barcodes, and poly-A tails (Fig. 4c). The cells are then encapsulated into a droplet with a bead containing oligo-dT primers. After cell lysis within the droplet, the bead captures both mRNAs and DNA barcodes conjugated to the antibodies through the binding of oligo-dT primers and poly-A tails, as described in scG&T-seq. A library is generated for mRNAs and proteins using RT and PCR amplification and is then sequenced to quantify both mRNAs and proteins. REAP-seq is a similar technology with a different construct of the barcode conjugated to the bead. Unlike the CITE- and REAP-seq methods, which target cell surface proteins, an alternative method, single-cell RNA and immunodetection (RAID), can detect intracellular proteins or phosphorylated proteins together with mRNAs^59^. After crosslinking and permeabilization, intracellular target proteins are immunostained in single cells using antibodies conjugated with RNA barcodes, which convert proteins into RNAs (Fig. 4d). After the cells are sorted into plates containing CEL-seq2-compatible primers, RNAs are liberated through reverse crosslinking and converted into cDNAs by RT. These methods can measure tens of proteins. Despite the limited proteome size, these methods are high throughput, allowing analysis of thousands of cells. The characteristics of these technologies are summarized in Table 1.

Numerous studies using these methods have been performed in diverse cellular systems to examine the link between the transcriptome and proteome at the single-cell level. For example, using the PEA method, Darmanis et al.^60^ investigated the effects of BMP4 on early-passage glioblastoma cells (U3035MG cell line) by measuring the levels of 82 mRNAs and 75 proteins (61 in common). They found subpopulations of glioblastoma cells showing distinct changes in mRNA and protein abundance after BMP4 treatment, suggesting significant heterogeneity in the response to BMP4. They further observed poor correlations between protein and mRNA expression levels across single cells, with proteins more accurately defining the response to BMP4. Moreover, using the PLAYR method, Frei et al.^56^ simultaneously quantified 40 different mRNAs and proteins, including cell surface markers, cytokines, and stem cell-related proteins, in several cell types, such as Jurkat T cells, NK cells, peripheral blood mononuclear cells, and embryonic stem cells. From the data obtained, they found that transcripts showed more gradual differences than the corresponding proteins (e.g., HLA-DRA) in single PBMCs and that proteins (e.g., ITGAX) were expressed even when there were virtually no corresponding transcripts in a subpopulation of PBMCs. Moreover, using the CITE-seq method, Stoeckius et al.^57^ captured 8005 cord blood mononuclear cells (CBMCs) using 13 well-characterized monoclonal antibodies that recognize cell surface proteins commonly used as markers for immune cell classification, and then characterized populations of CBMCs based on mRNA profiles measured from the single CBMCs. CITE-seq has recently been modified to expanded CRISPR-compatible cellular indexing of transcriptomes and epitopes by sequencing (ECCITE-seq), which provides multiple modalities of information, including transcriptome, protein, clonotype, and CRISPR perturbation data, with high sensitivity at a single-cell resolution^61^.

## Methods for single-cell omics data analysis

Single-cell mono-omics analysis provides different types of information, including data on genomic alterations (mutations and CNVs), DNA methylation sites, open chromatin sites, and mRNA or protein abundance, at the single-cell level. Different methods have been developed for each type of data to achieve diverse goals based on the corresponding information. For scRNA-seq data, various methods have been developed to identify cell populations, regulatory networks, and cellular trajectories^62^. First, for cell population characterization, the methods cluster cells based on the similarity of the expression profile and identify marker genes that are predominantly expressed in each cell cluster. For cell clustering, many methods combine dimensionality reduction and clustering analysis. Principal component analysis^63^ and t-distributed stochastic neighbor embedding^64^ have been widely used for dimensionality reduction. Recently, methods that deal with missing values^65^ or neural network-based models^66^ have been developed. Clustering methods differ in distance measures (e.g., Euclidean distance and inverted correlation), clustering algorithms (e.g., k-means, hierarchical, and graph-based clustering), and the capability to perform simultaneous clustering of genes and cells. The frequently used methods include Seurat^67^, pcaReduce^68^, SC3^69^, BackSPIN^70^, and SNN-cliq^71^. The detailed algorithms and applications of these and other methods have been extensively reviewed^72–74^.

Second, another set of methods infer the regulatory networks delineating regulatory relationships among marker genes (e.g., transcription factors and their targets) showing coexpression across different cells in a cell population. Although scRNA-seq offers many advantages in network inference over bulk RNA-seq, the zero inflation caused by dropout events and the high dimensionality of scRNA-seq data make it difficult to correctly infer regulatory networks under this approach. To address these issues, network inference methods generate appropriate models for zero-inflated distributions and infer network models on the basis of discretized regulatory relationships (e.g., binary values) or cell populations and gene modules with similar expression profiles. The methods that are frequently used for network inference include the SCNS toolkit^75^, inferenceSnapshot^76^, SCODE^77^, and SCENIC^78^ approaches. The detailed algorithms and applications of these and other methods have been extensively reviewed^79,80^. Finally, there is a set of methods for inferring the cellular trajectory (e.g., the differentiation trajectory) describing the temporal evolution of cells, which is estimated through the transition analysis of expression profiles. Early trajectory inference methods fixed the topology of the trajectory (e.g., linear, bifurcated, or cyclic) and focused on correctly ordering the cells along the fixed topology. However, recently developed methods infer the topology of the trajectory and the order of cells on the individual branches at the same time. The methods that are frequently used for cell trajectory inference include Monocle^81^, DPT^82^, Wishbone^83^, and Waddington-OT^84^. These and other methods have been extensively reviewed by Saelens et al.^85^.

For scWGS data, the major goals are to identify CNVs and single-nucleotide variations (SNVs) at the single-cell level^54^. To achieve these goals, the algorithms developed to identify CNVs in bulk WGS data have been modified to effectively handle the low coverage of the genome found in scWGS data^86^. Various methods for identifying CNVs from scWGS data have been developed, including Ginkgo^87^, baseqCNV^88^, SCNV^89^, SCCNV^90^, and SCOPE^91^. To identify the regions of copy number gains or losses, these methods use the segmentation procedure of circular binary segmentation (CBS). Moreover, several methods, such as SCcaller^92^, baseqSNV^88^, MonoVar^93^, and SCAN-SNV^94^, have been developed to effectively identify SNVs from scWGS data with high allele coverage biases due to the low coverage of the genome and high PCR amplification error. For example, for a given SNV, SCcaller^92^ estimates the distribution of the allele fractions of heterozygous germline single-nucleotide polymorphisms in the region containing the SNV as a model of the local allele bias and then adjusts the probability of the SNV based on the local allele bias model. These and other methods have been extensively reviewed^1^.

For single-cell epigenome data, the major goals are to identify open chromatin and DNA methylation sites in single cells. Single-cell epigenome analysis produces a low depth of DNA sequences compared to bulk analysis, making it difficult to identify the peaks corresponding to open chromatin or DNA methylation sites. One strategy for resolving this problem is to aggregate the data from ~100 single cells, identify peaks using the algorithms developed for bulk data, and then determine whether these peaks are present in each single cell using the data from the single cells. scABC^95^ uses this strategy to identify open chromatin sites from scATAC-seq data. Nevertheless, this strategy can still miss peaks corresponding to a low level of DNA methylation or open chromatin due to a lack of information even in aggregated data. Another strategy is to aggregate signals from adjacent regions or regions with similar regulatory elements. For example, Smallwood et al.^41^ binned the genome into segmented regions and then identified the regions with high read counts as regions including putative peaks for DNA methylation sites. Farlik et al.^36^ identified the regions including peaks by combining the data for regions sharing DNA methylation according to a cis-regulatory element database such as encyclopedia of DNA elements (ENCODE). Among these methods, chromVAR^96^ uses this strategy to identify open chromatin sites from scATAC-seq data, while cisTopic^97^ and SCALE^98^ combine the results from cell- and region-level aggregation for peak identification. These and other methods have been extensively reviewed^99,100^.

## Integrative analysis of single-cell multiomics data

**Fig. 5:**
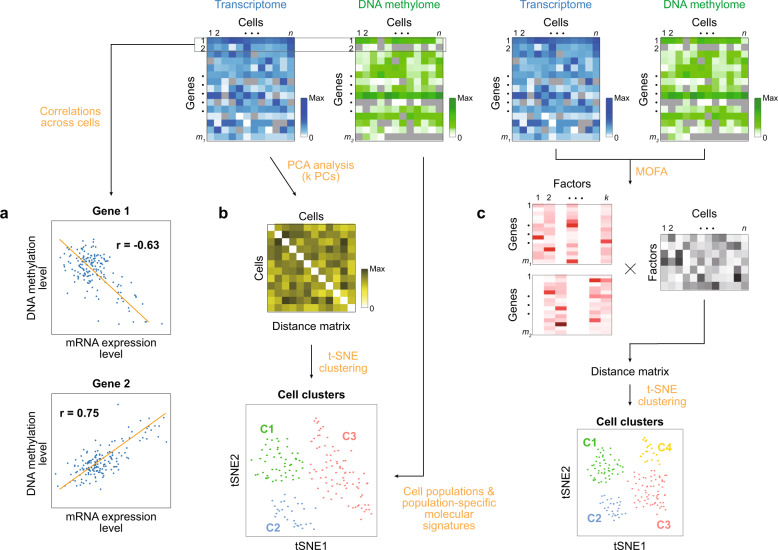
Strategies for the integrative analysis of single-cell multiomics data. Blue and green heat maps represent the data matrixes for the transcriptome and DNA methylome, respectively. The symbols *n*, *m*_*1*_, and *m*_*2*_ denote the numbers of cells (*n*) and genes with the levels of mRNA (*m*_*1*_) and DNA methylation (*m*_*2*_). Colors in the heat maps represent the levels of mRNA and DNA methylation (see color bars; Max, the maximum level). **a** Correlation analysis between mRNA and DNA methylation levels. Scatter plots show mRNA and DNA methylation levels for genes 1 (top) and 2 (bottom). Line, regression line; *r*, Pearson correlation. Negative and positive correlations are shown for genes 1 and 2, respectively. **b** Analysis of scRNA-seq data followed by the integration of scBS-seq data. Principal component analysis (PCA) is first applied to scRNA-seq data to obtain score values for *k* PCs, the pairwise Euclidean distances of cells are computed using the score values for *k* PCs to generate a distance matrix, t-stochastic neighbor embedding (t-SNE) clustering is applied to the distance matrix to identify cell populations, and scBS-seq data are then integrated into these cell populations as described in the text. C1-3, cell populations 1-3, respectively. **c** Integrative analysis of scRNA-seq and scBS-seq data to generate the overall single-cell map. The analytical scheme of MOFA is shown. Two-way matrix decomposition is performed for scRNA-seq and scBS-seq data using *k* factors, resulting in weight matrixes (*m*_*1*_ × *k* for scRNA-seq data and *m*_*2*_ × *k* for scBS-seq data) and a factor loading matrix (*k* × *n* for *n* cells). Factor loading values are used to compute a distance matrix that is then used for t-SNE clustering. The t-SNE plot shows cell populations 1-4 (C1-4) identified collectively by scRNA-seq and scBS-seq data.

For the integrative analysis of single-cell multiomics data, the methods developed for single-cell mono-omics data have been extended and combined. The strategies can be categorized as (1) correlation analysis between single-cell mono-omics data (Fig. 5a); (2) the analysis of one type of single-cell data (e.g., scRNA-seq) followed by the integration of another single-cell data type (e.g., SNVs from scWGS or open chromatin sites from scATAC-seq) (Fig. 5b); and (3) the integrative analysis of all types of single-cell omics data to generate the overall single-cell map (e.g., for a cell population or differentiation trajectory) (Fig. 5c).

A number of studies have used the first strategy to examine the correlation of CNVs^24^ or DNA methylation levels^39,40^ with mRNA expression levels at the single-cell level. For example, Angermueller et al.^39^ applied scM&T-seq to mouse embryonic stem cells and computed the weighted Pearson correlations of DNA methylation levels in several genomic contexts (promoters, distal regulatory elements, and gene bodies) with mRNA expression levels across single cells for individual genes. Negative correlations of DNA methylation and mRNA expression levels were found to be dominant in non-CpG island promoters, whereas both positive and negative correlations were observed for distal regulatory elements. Correlation analysis has also been applied to examine the relationship between mRNA and protein expression levels^58^. For example, Peterson et al.^58^ applied REAP-seq to PBMCs and computed the Pearson correlations between mRNA and protein expression levels across single cells for immune cell markers. They found that the levels of mRNAs and proteins were poorly correlated and that protein quantification was more sensitive than mRNA quantification for markers with low mRNA expression.

Under the second strategy, scRNA-seq is the most common single-cell mono-omics data type into which the other data are integrated, due to its higher coverage of the transcriptome compared to those provided by other single-cell omics data types; scWGS, scBS-seq or scATAC-seq data exhibit low coverage of the genome, while CITE-seq data exhibit lower coverage of the whole proteome. For example, Cao et al.^49^ applied sci-CAR to mouse kidney cells and classified 10,727 cells into 14 subpopulations using scRNA-seq data. They further identified open chromatin sites (total 22,026 sites) unique to each of the 14 subpopulations and identified cis-regulatory elements (e.g., transcription factor binding sites) that may contribute to the population-specific expression of several marker genes. Moreover, Stoeckius et al.^57^ applied CITE-seq to CBMCs and identified 15 populations of 8005 cells using scRNA-seq data based on 556 genes showing population-specific expression. Scatter plot analyses for every pair of antibodies using tag counts derived from the antibodies showed that protein expression levels could be used to further subdivide cell populations identified from scRNA-seq with subtle mRNA expression differences, such as the NK cell population.

The third strategy is commonly used when the different single-cell omics data being integrated present comparable coverage. Otherwise, integration may lead to bias toward the data with a higher coverage. For this third strategy, several matrix factorization-based methods have recently been developed, including linked inference of genomic experimental relationships (LIGER)^101^ and multi-omics factor analysis (MOFA)^102^. LIGER employs integrative nonnegative matrix factorization (iNMF) for integration. Although this method has been applied to integrate scRNA-seq and scBS-seq data from different single-cell mono-omics analyses, it may be applicable to multiple datasets from single-cell multiomics data. LIGER defines cell populations according to genes for which (1) both mRNA expression and DNA methylation levels are available or (2) only either mRNA expression or DNA methylation data are available. For the former cell populations, the relationships between mRNA expression and DNA methylation can reveal the potential regulatory effects of DNA methylation on mRNA expression for the genes defining these populations. MOFA employs a multiway matrix decomposition method that generates one factor (cell population) loading matrix (the degree to which cells can belong to the individual cell populations) and one weight matrix for each data type (the degree to which molecular features can contribute to the cell populations according to the individual data)^39^. MOFA was applied to previously reported mRNA expression and DNA methylation data generated from mouse embryonic stem cells by scM&T-seq after the imputation of missing values in the individual datasets. MOFA provided the genes whose mRNA expression and/or DNA methylation levels contributed greatly to each cell population, thereby enabling the regulatory relationships between mRNA and DNA methylation defining the cell population to be inferred. The authors further determined a differentiation trajectory of mouse embryonic stem cells in the factor space and then identified the genes showing mRNA expression and DNA methylation patterns associated with the transition along the trajectory based on the molecular features defining the factors in the weight matrixes^102^.

## Discussion

Single-cell multiomics approaches provide unprecedented opportunities to systematically explore cellular diversity and heterogeneity by enabling a more comprehensive delineation of the state of single cells than that provided by single omics data based on multichannel molecular readouts. The integration of multiple molecular readouts can provide insights into causal factors that regulate cellular states based on the associations between causal factors and target genes in cell populations. For example, genotype–phenotype correlations measured via the integrative analysis of single-cell genome and transcriptome data can reveal the link between genomic alterations and the transcriptional consequences for target genes involved in disease-related processes. Furthermore, the integrative analysis of the epigenome and transcriptome can provide regulatory links between epigenetic changes and the expression of target genes. In addition, the integration of information from multiple omics layers, including DNA, RNA, and protein data, can enhance the accuracy of the identification of cell populations, cellular trajectories, or lineage tracing and new or rare cell types.

The applications of single-cell multiomics analyses are still at an early stage. There remain many paths to be explored and considerable opportunities for expansion. Moreover, there are still several technical and computational limitations that should be overcome to improve both the content and the quality of the information obtained from single-cell multiomics analysis. For example, bisulfite treatment can lead to DNA damage that can affect the accuracy of the measured DNA methylome. Additionally, cell fixation is prone to reduce the yield of information, thereby introducing bias in the measurements. Furthermore, the number of proteins that can be detected using current single-cell multiomics technologies is limited due to insufficient sensitivity, which may introduce bias in the interpretation of the proteome because the functions of the measured proteins in single cells should be defined by their interactions with other proteins. The optimization of existing single-cell experimental protocols or development of new protocols is also required to improve the sensitivity (mutations, CNVs, and proteomes), accuracy (DNA methylation and phosphoproteome), and coverage (mutations, CNVs, and proteomes) of single-cell multiomics measurements. Moreover, new omics combinations for different multichannels of molecules (e.g., integrative analysis of single-cell genome and proteome) are needed to infer unprecedented regulatory associations (e.g., the mutation and phosphorylation of signaling molecules).

Despite the rich resources of experimental protocols available for single-cell omics analysis, computational methods for the integrative analysis of single-cell multiomics data have just begun to emerge. While recent advances in scRNA-seq technologies have led to an exponential increase in the number of cells and genes probed, the technologies available for other types of single-cell omics analyses still provide intrinsically sparse information with a significant fraction of missing values and high levels of noise signals. Thus, methods that can effectively handle the discrepancy in the coverage of information between the transcriptome and other types of single-cell omics data are needed for more sophisticated multiomics statistical models during data integration. In addition, given such missing values, systematic noise, and coverage discrepancy, the improvement of existing data analysis methods or development of new data analysis methods is necessary to optimally extract information through the integrative analysis of single-cell multiomics data. Furthermore, most of the current methods used for single-cell multiomics analyses are limited to the integration of two omics layers at once. As single-cell multiomics technologies for measuring more omics layers emerge, methods that can integrate three or more types of omics data are required for the effective characterization of regulatory relationships among the different omics layers.

Advancements in both experimental technologies and data analysis methods for single-cell multiomics analysis are critical to ensure more accurate regulatory relationships among different omics layers for important molecules in disease pathogenesis. These regulatory relationships can provide new insights into the molecular mechanisms underlying disease-related processes at the single-cell level, as illustrated by the integrative analysis of multiple bulk omics data^7–9,13^. These molecular mechanisms can then reveal new diagnostic markers and therapeutic targets, thereby transforming the current strategies for the diagnosis and therapy of diseases.
